# Supplementary figures and images for: Across-breed analyses of genome-wide association studies for stature and mammary gland morphology in cattle reveal pleiotropic effects of the Friesian POLLED haplotype
Source: Genet Sel Evol. 2026 Mar 11;58:19. doi: 10.1186/s12711-026-01042-z (PMC12983537; doi:10.1186/s12711-026-01042-z)

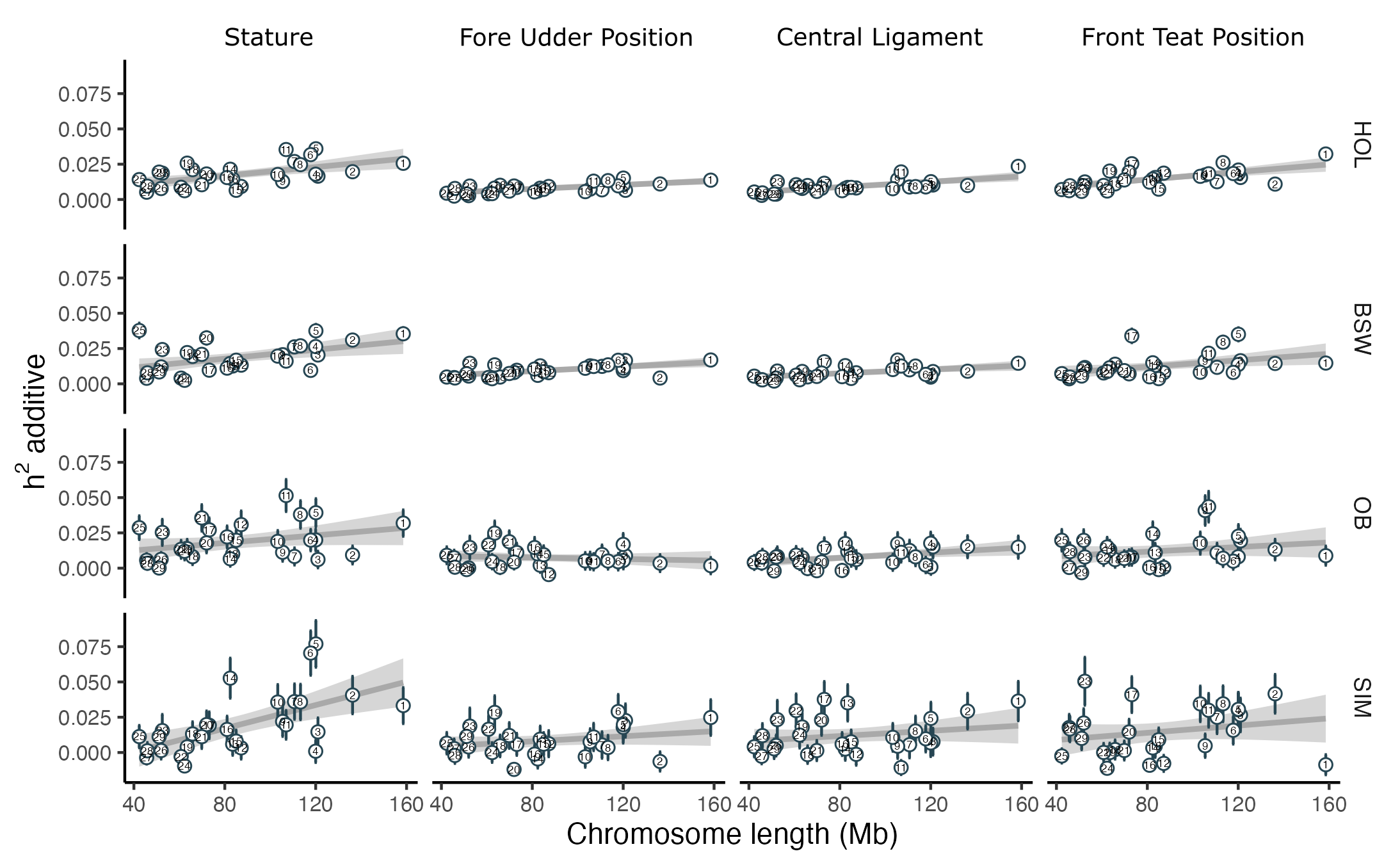

Supplement: Supplementary file 2 — Supplementary Material 2: Figure S1. Additive SNP-based heritability (h2) per chromosome across four breeds for stature and mammary gland morphology traits. Additive h2 partitioned across chromosomes plotted against chromosome length (Mb). Breeds included were Brown Swiss (BSW), Original Braunvieh (OB), Holstein (HOL) and Simmental (SIM). Traits are stature with height at sacral bone, and mammary gland morphology traits are fore udder position, front teat position and udder central ligament. Vertical lines outside the circles indicate the standard errors. The black line is a regression line, and the grey shaded area represents the 95% confidence interval. [file 12711_2026_1042_MOESM2_ESM.png]

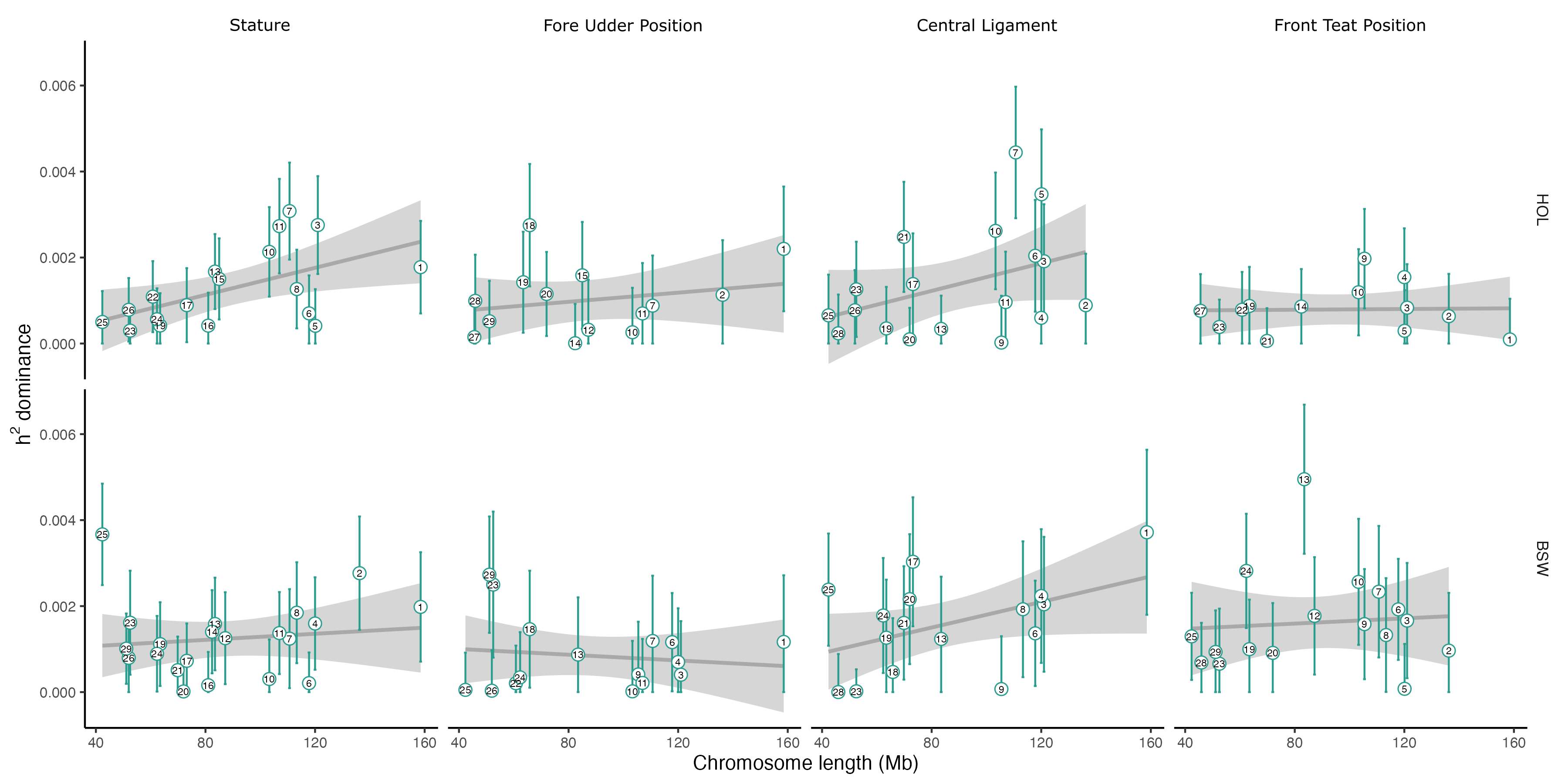

Supplement: Supplementary file 3 — Supplementary Material 3: Figure S2. Dominance SNP-based heritability (h2SNP) per chromosome for two breeds for stature and mammary gland morphology traits. Dominance h2 (from additive and dominance h2 estimation) partitioned across chromosomes, plotted against chromosome length (Mb). Breeds included were Brown Swiss (BSW) and Holstein (HOL). Traits are stature with height at sacral bone (KBHM), and the mammary gland morphology traits are fore udder position, front teat position and udder central ligament. Vertical lines outside the circles indicate the standard errors. The black line is a regression line, and the grey shaded area represents the 95% confidence interval. [file 12711_2026_1042_MOESM3_ESM.png]

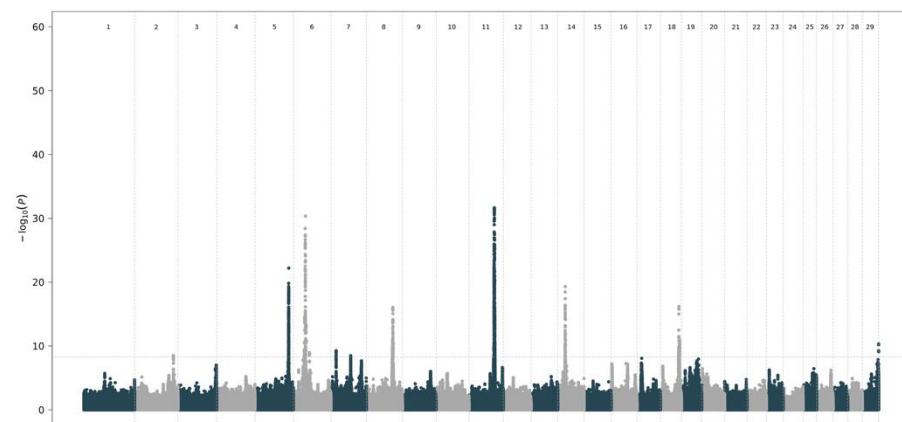

HOL

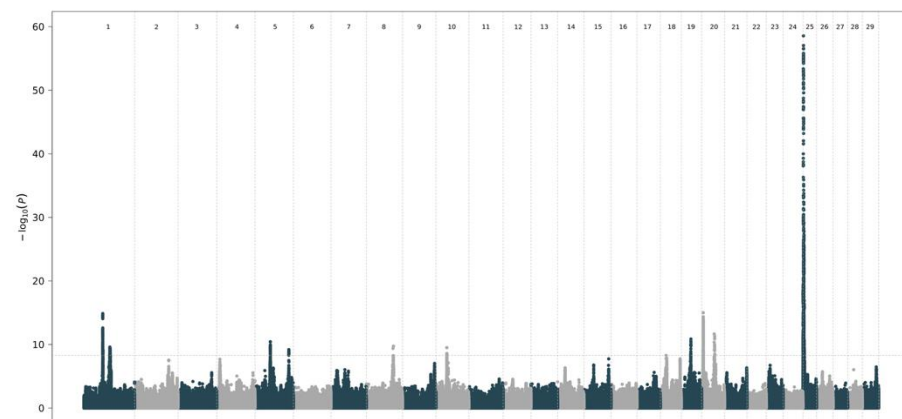

BSW

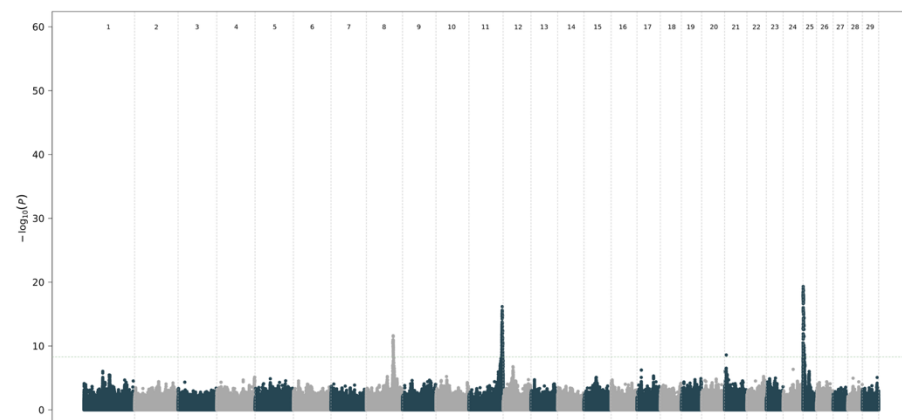

OB

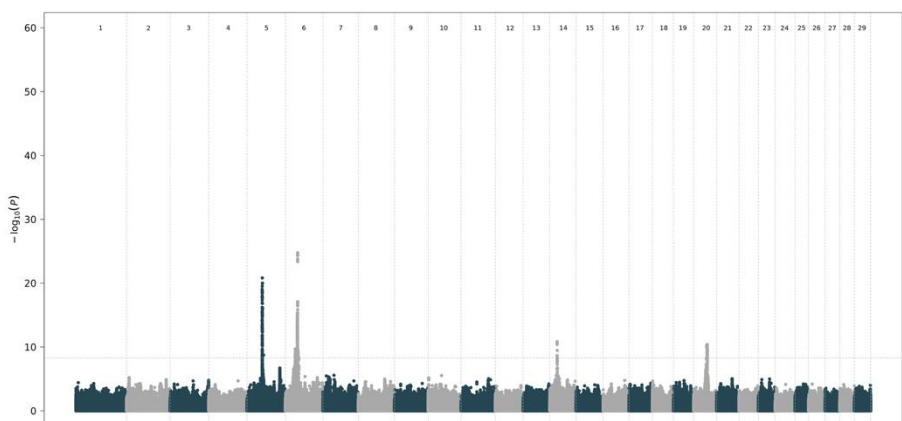

SIM

Supplement: Supplementary file 4 — Supplementary Material 4: Figure S3. Additive genome-wide association studies for stature across four breeds. Breeds included were Brown Swiss (BSW), Original Braunvieh (OB), Holstein (HOL) and Simmental (SIM). The –log10(p) values are plotted against the genomic position by chromosome. The horizontal line denotes the genome-wide significance threshold (p < 5e−09). [file 12711_2026_1042_MOESM4_ESM.pdf]

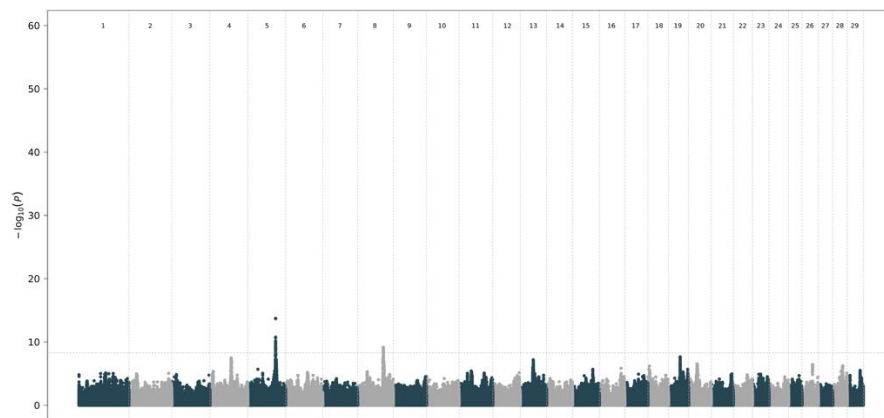

HOL

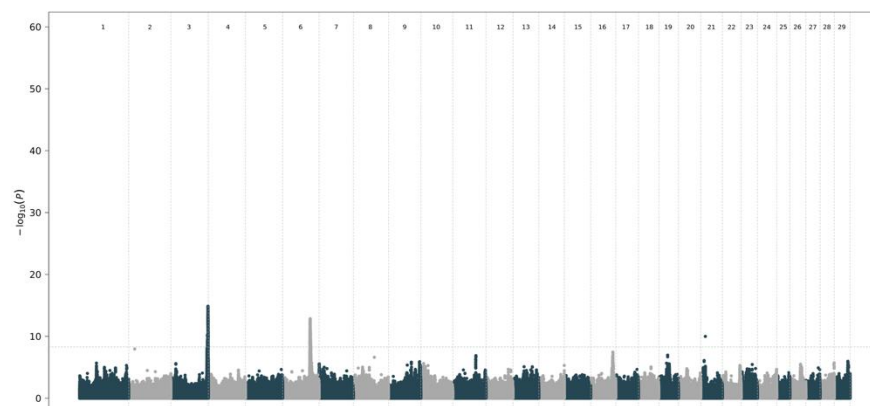

BSW

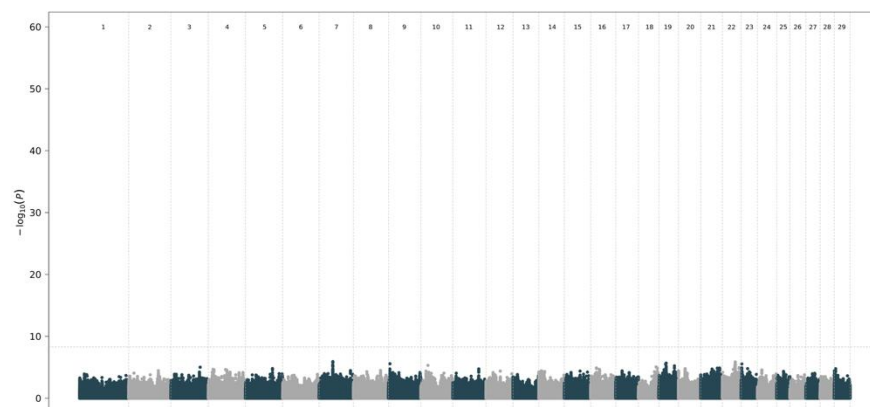

OB

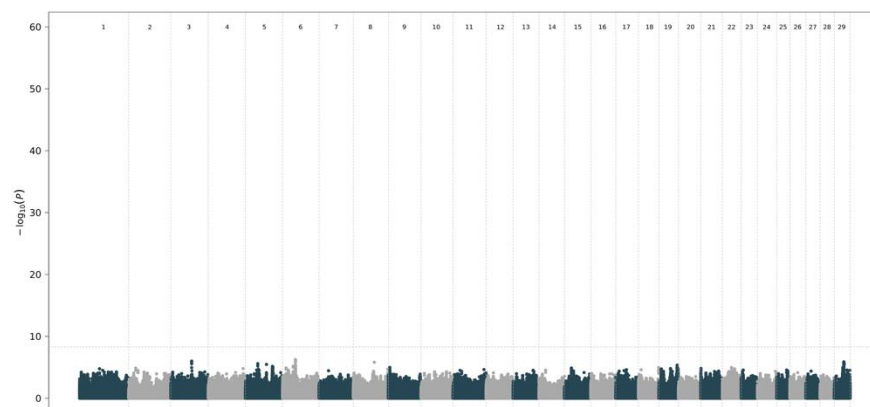

SIM

Supplement: Supplementary file 5 — Supplementary Material 5: Figure S4. Additive genome-wide association studies for fore udder position across four breeds. Breeds included were Brown Swiss (BSW), Original Braunvieh (OB), Holstein (HOL) and Simmental (SIM). The –log10(p) values are plotted against genomic position by chromosome. The horizontal line denotes the genome-wide significance threshold (p < 5e−09). [file 12711_2026_1042_MOESM5_ESM.pdf]

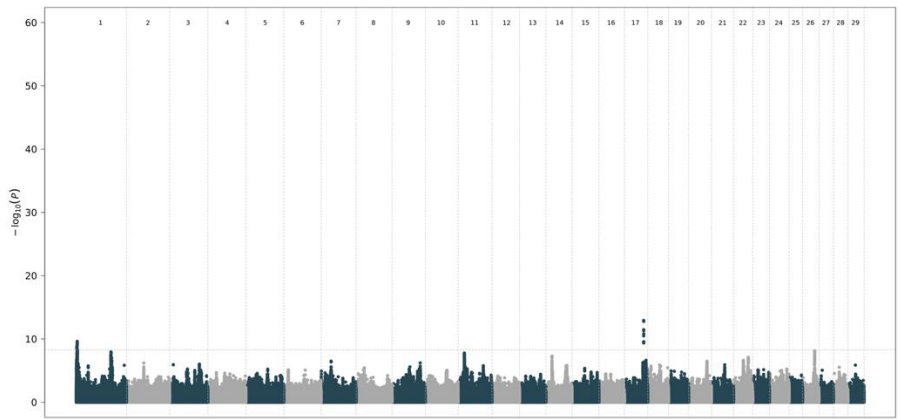

HOL

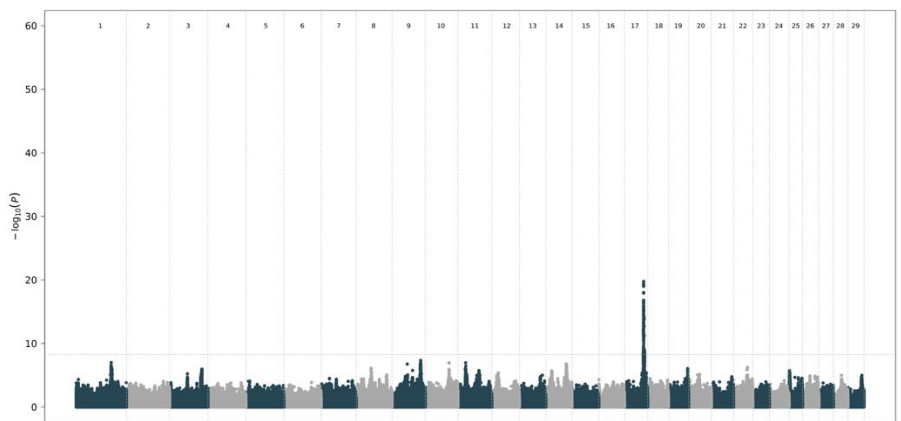

BSW

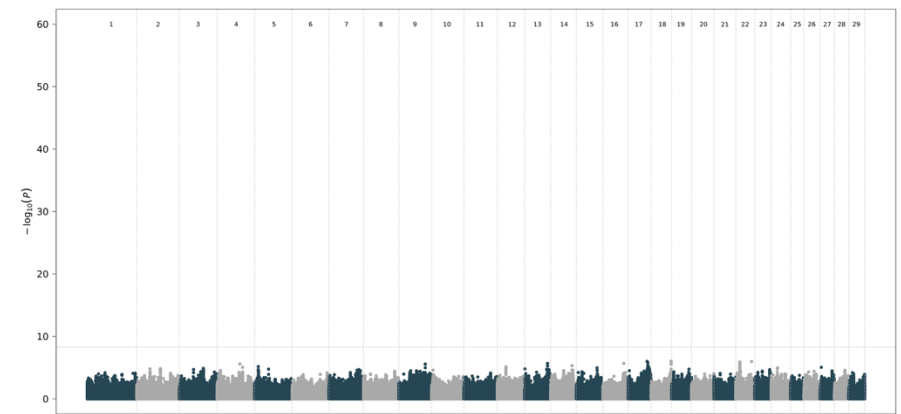

OB

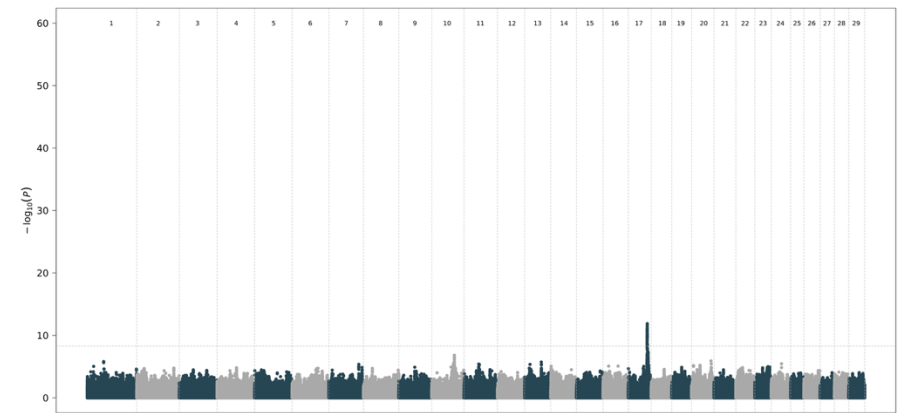

SIM

Supplement: Supplementary file 6 — Supplementary Material 6: Figure S5. Additive genome-wide association studies for udder central ligament across four breeds. Breeds included were Brown Swiss (BSW), Original Braunvieh (OB), Holstein (HOL) and Simmental (SIM). The –log10(p) values are plotted against genomic position by chromosome. The horizontal line denotes the genome-wide significance threshold (p < 5e−09). [file 12711_2026_1042_MOESM6_ESM.pdf]

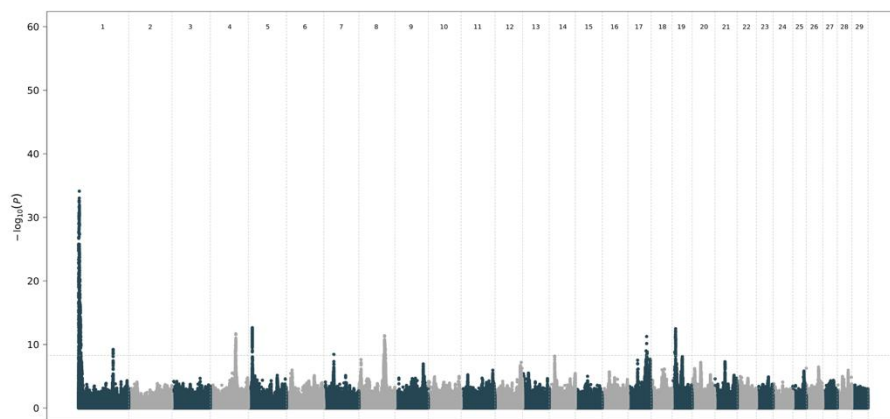

HOL

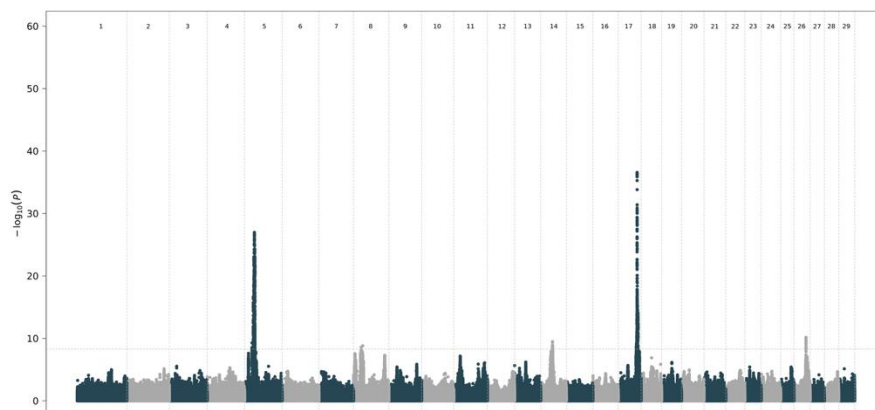

BSW

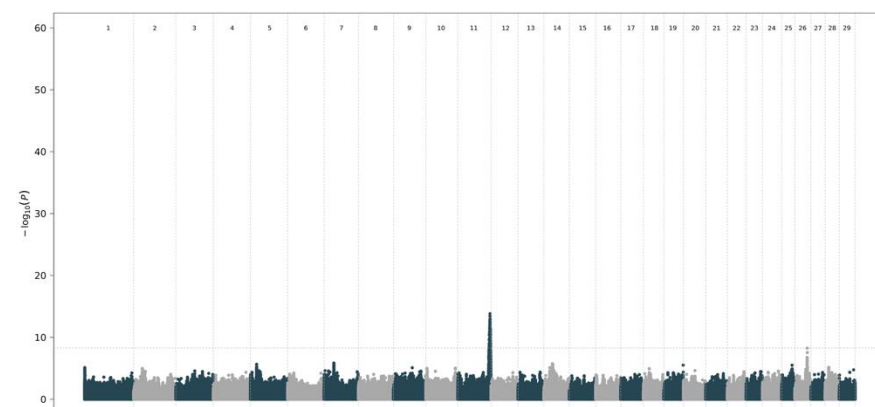

OB

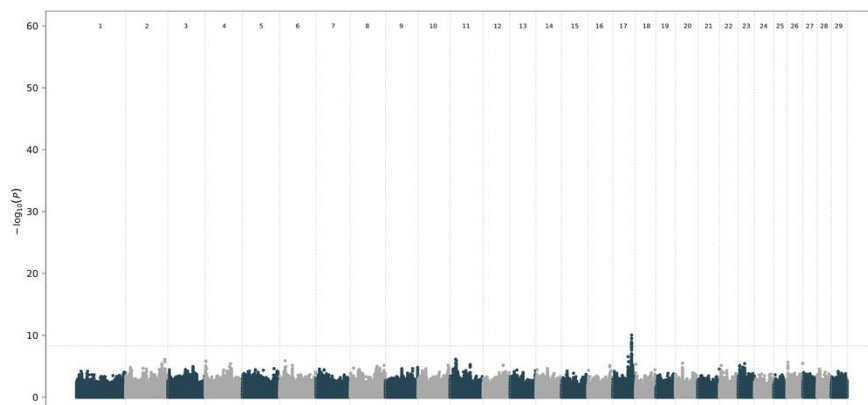

SIM

Supplement: Supplementary file 7 — Supplementary Material 7: Figure S6. Additive genome-wide association studies for front teat position across four breeds. Additive genome-wide association test for udder central ligament across four breeds. Breeds included were Brown Swiss (BSW), Original Braunvieh (OB), Holstein (HOL) and Simmental (SIM). The –log10(p) values are plotted against genomic position by chromosome. The horizontal line denotes the genome-wide significance threshold (p < 5e−09). [file 12711_2026_1042_MOESM7_ESM.pdf]

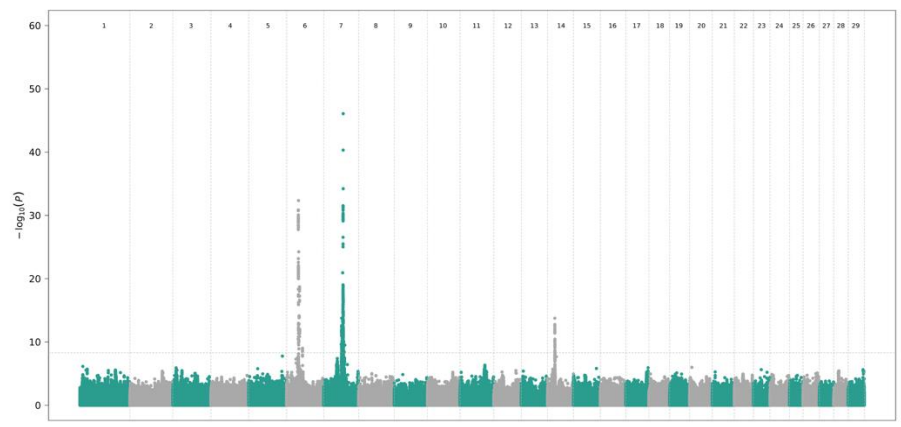

HOL

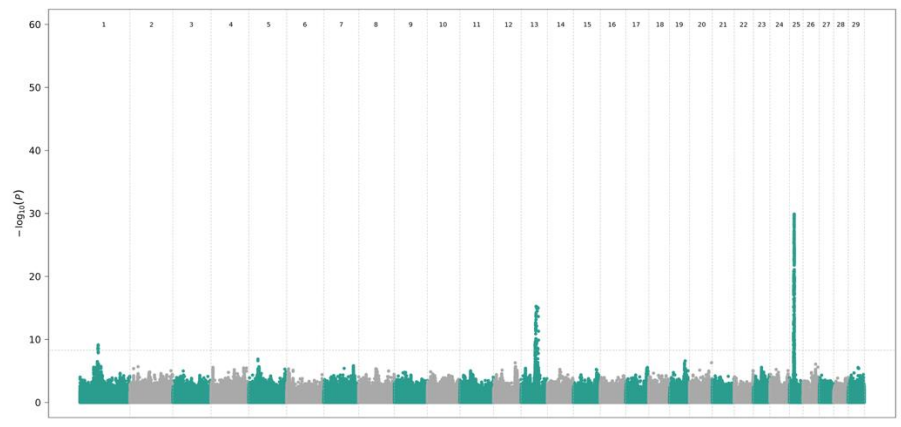

BSW

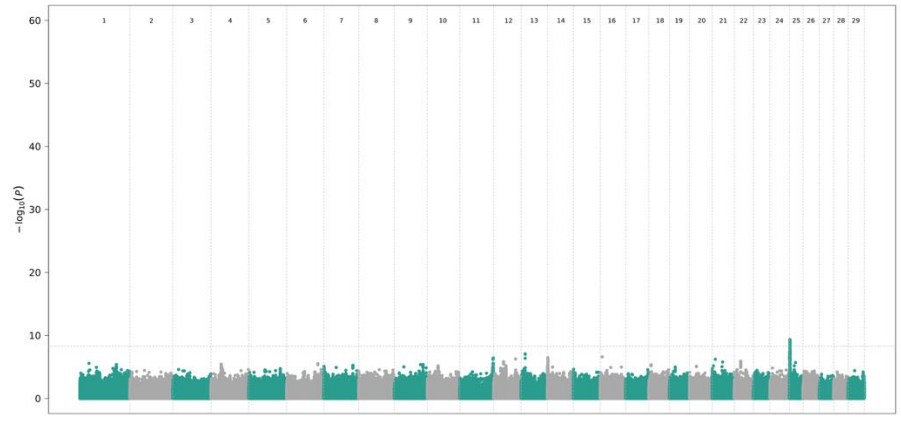

OB

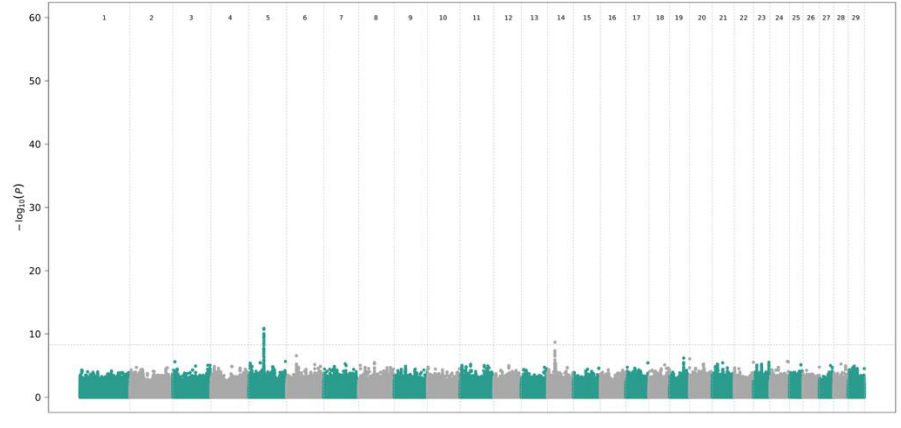

SIM

Supplement: Supplementary file 8 — Supplementary Material 8: Figure S7. Dominance genome-wide association studies for stature across four breeds. Breeds included were Brown Swiss (BSW), Original Braunvieh (OB), Holstein (HOL) and Simmental (SIM). The –log10(p) values are plotted against genomic position by chromosome. The horizontal line denotes the genome-wide significance threshold (p < 5e−09). [file 12711_2026_1042_MOESM8_ESM.pdf]

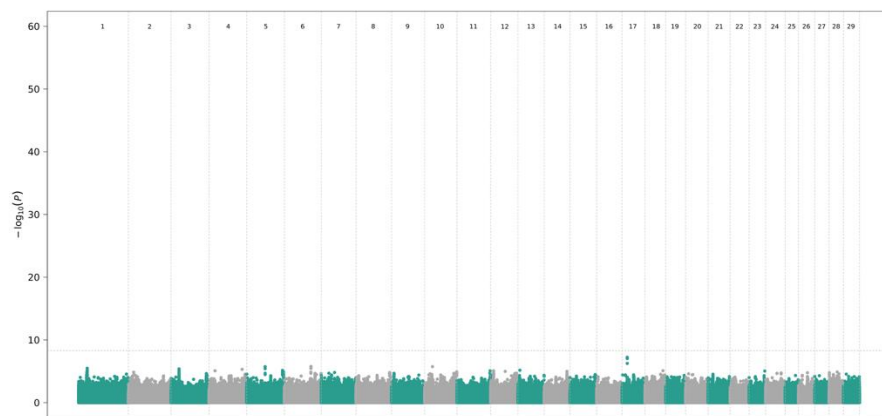

HOL

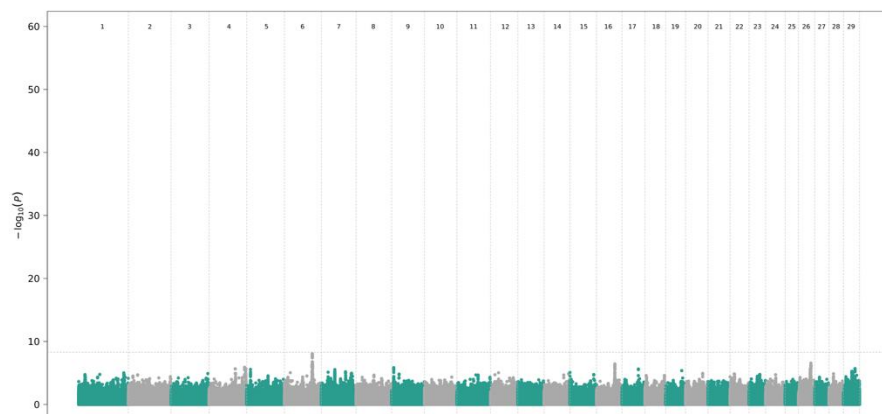

BSW

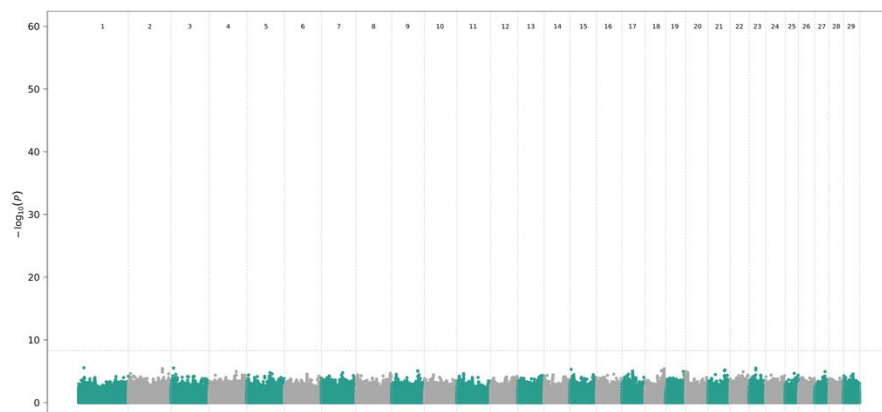

OB

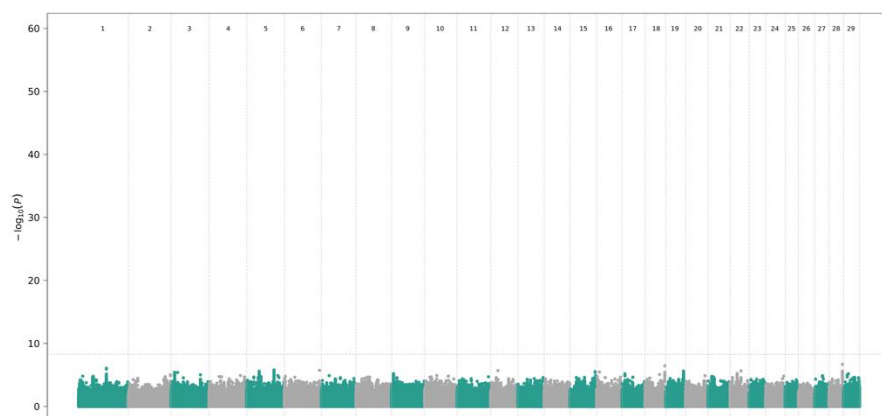

SIM

Supplement: Supplementary file 9 — Supplementary Material 9: Figure S8. Dominance genome-wide association studies for fore udder position across four breeds. Breeds included were Brown Swiss (BSW), Original Braunvieh (OB), Holstein (HOL) and Simmental (SIM). The –log10(p) values are plotted against genomic position by chromosome. The horizontal line denotes the genome-wide significance threshold (p < 5e−09). [file 12711_2026_1042_MOESM9_ESM.pdf]

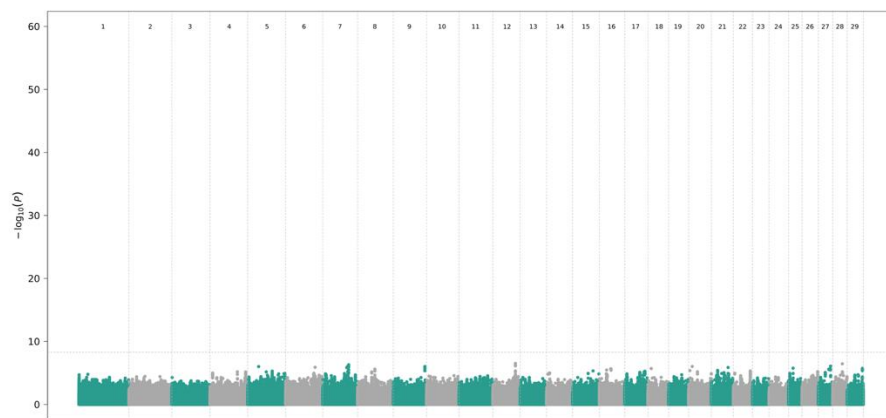

HOL

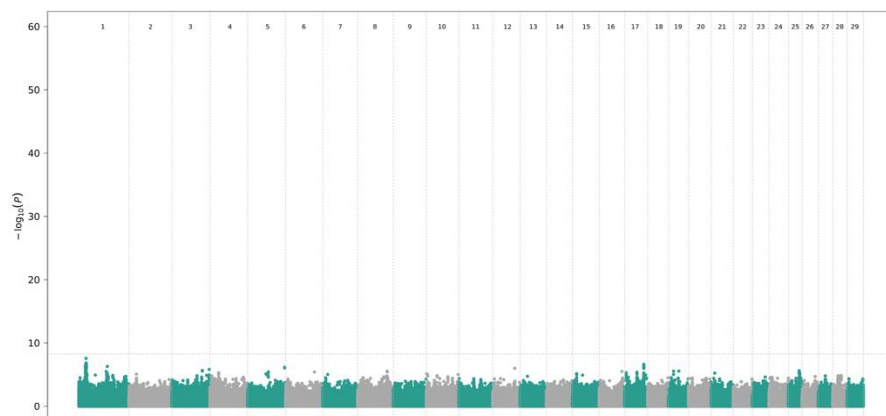

BSW

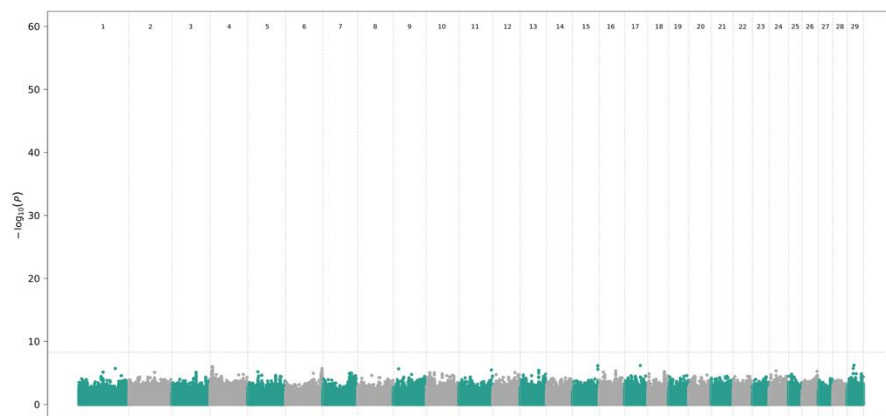

OB

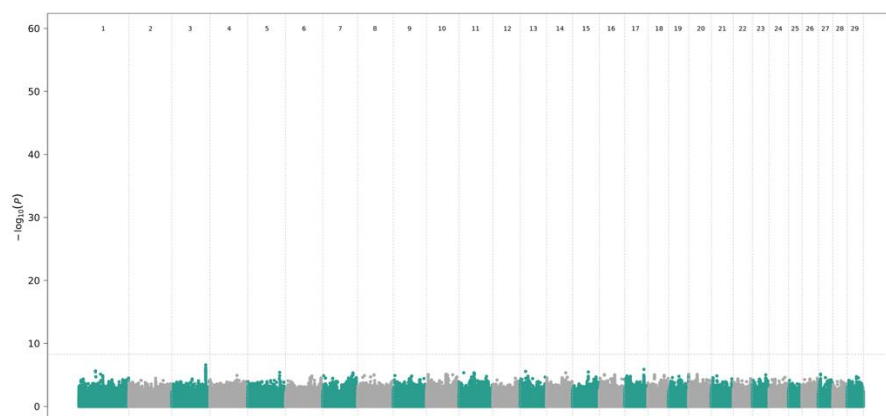

SIM

Supplement: Supplementary file 10 — Supplementary Material 10: Figure S9. Dominance genome-wide association studies for udder central ligament across four breeds. Breeds included were Brown Swiss (BSW), Original Braunvieh (OB), Holstein (HOL) and Simmental (SIM). The –log10(p) values are plotted against genomic position by chromosome. The horizontal line denotes the genome-wide significance threshold (p < 5e−09). [file 12711_2026_1042_MOESM10_ESM.pdf]

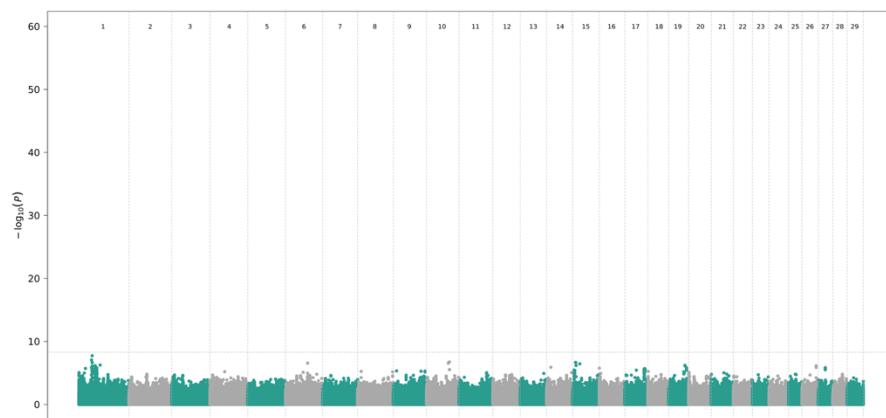

HOL

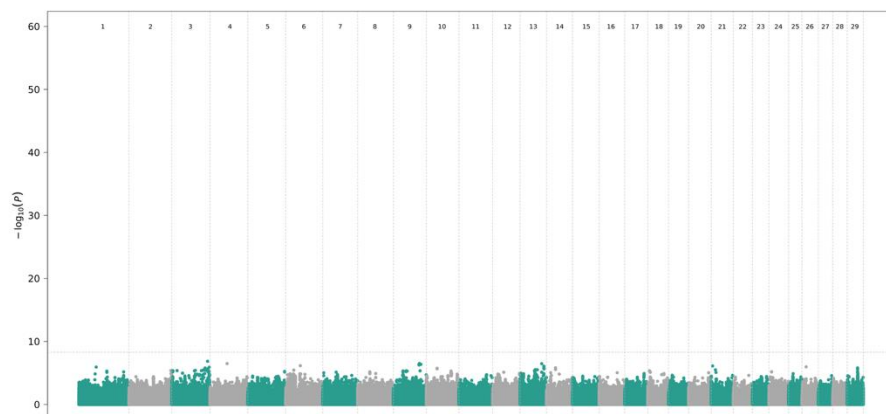

BSW

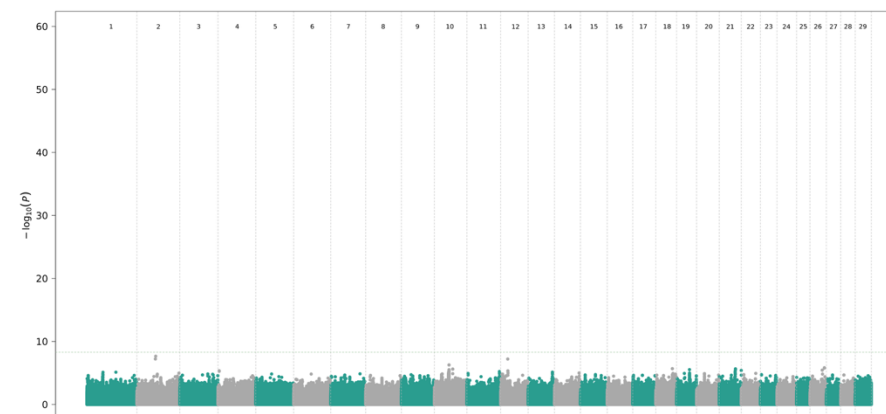

OB

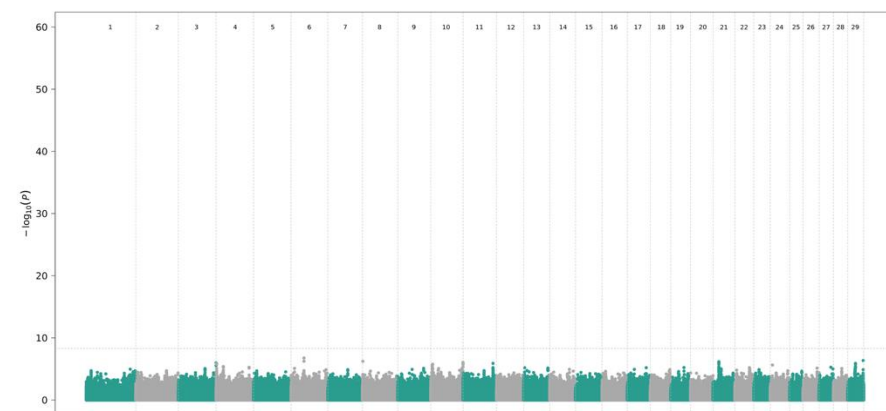

SIM

Supplement: Supplementary file 11 — Supplementary Material 11: Figure S10. Dominance genome-wide association studies for front teat position across four breeds. Breeds included were Brown Swiss (BSW), Original Braunvieh (OB), Holstein (HOL) and Simmental (SIM). The –log10(p) values are plotted against genomic position by chromosome. The horizontal line denotes the genome-wide significance threshold (p < 5e−09). [file 12711_2026_1042_MOESM11_ESM.pdf]

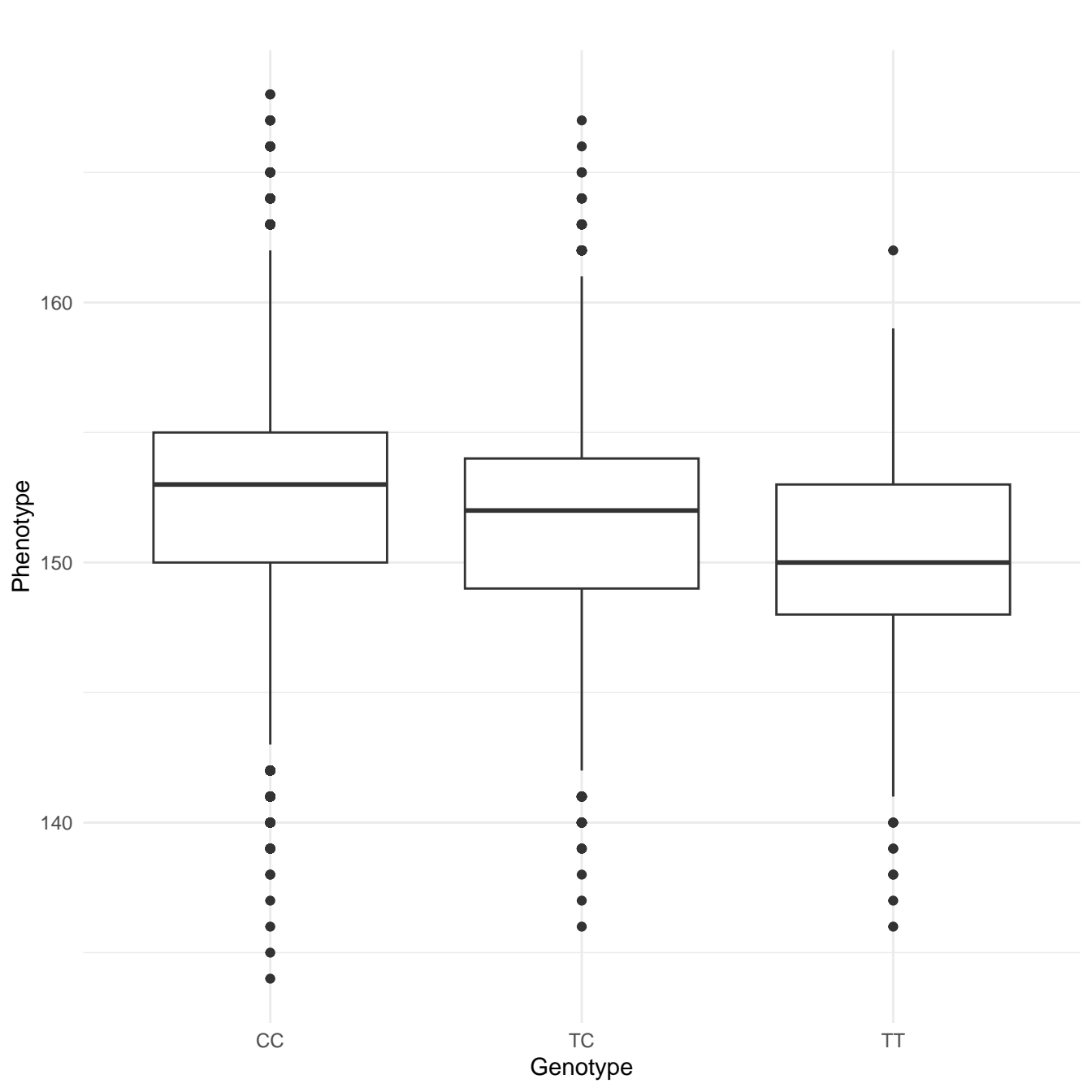

Supplement: Supplementary file 13 — Supplementary Material 13: Figure S12. A stature QTL on BTA7 in Holstein cattle. Effect of chr7:61828471 on stature in Holstein cattle. This marker reached genome-wide significance under the dominance model (p = 8.38e−47). Phenotype distributions are presented for each genotype class. [file 12711_2026_1042_MOESM13_ESM.pdf]

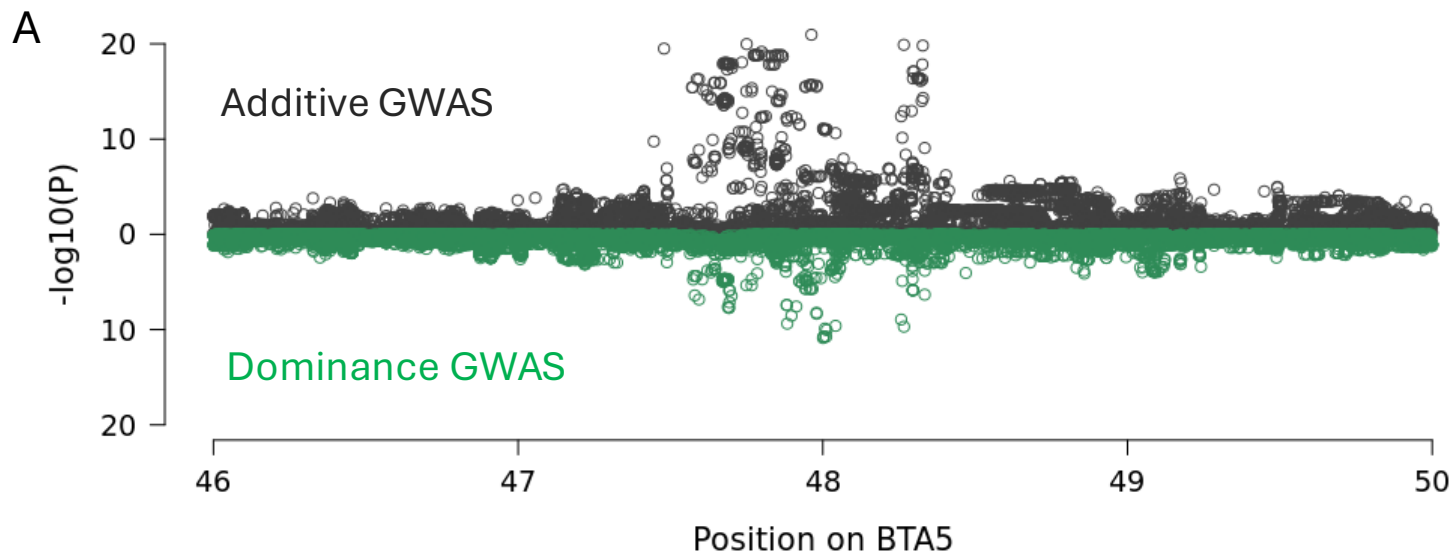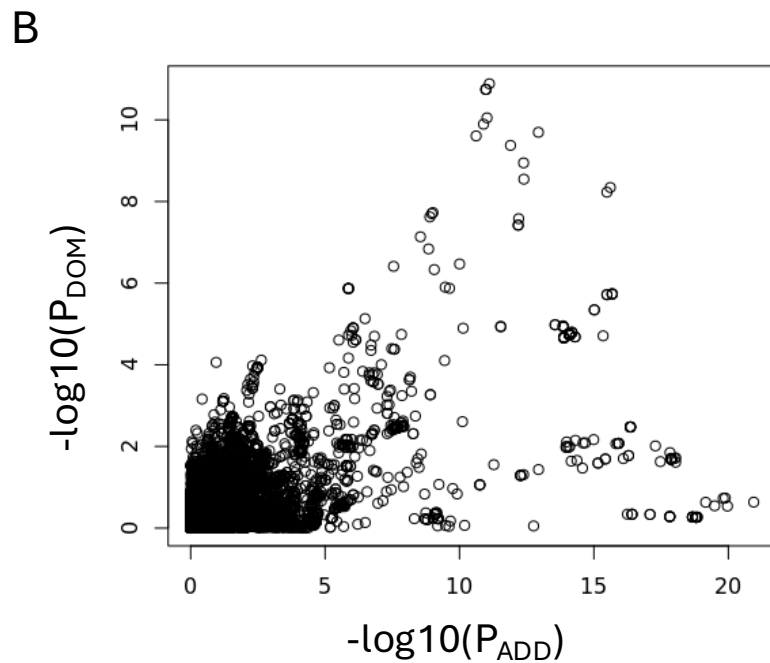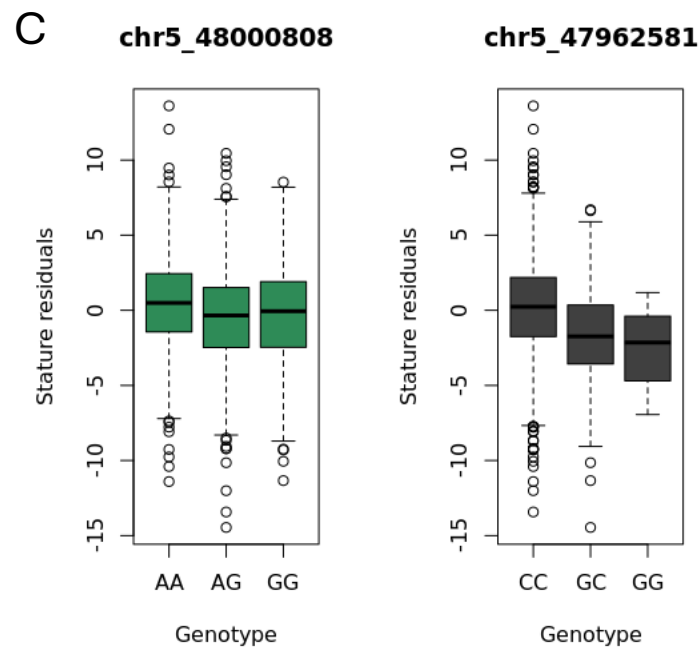

Supplement: Supplementary file 14 — Supplementary Material 14: Figure S13. A stature QTL on BTA5 in Simmental cattle. A Association testing with the additive (grey) and dominant (blue) model indicate the presence of QTL at approximately 48 Mb on bovine chromosome 5 encompassing the HMGA2 gene. B Scatterplot comparing the association (−log10(P)) of variants from the additive (ADD) and dominance (DOM) model. C Boxplots representing the effects of the top variants from the dominance (green) and additive (grey) models on stature. [file 12711_2026_1042_MOESM14_ESM.pdf]

A

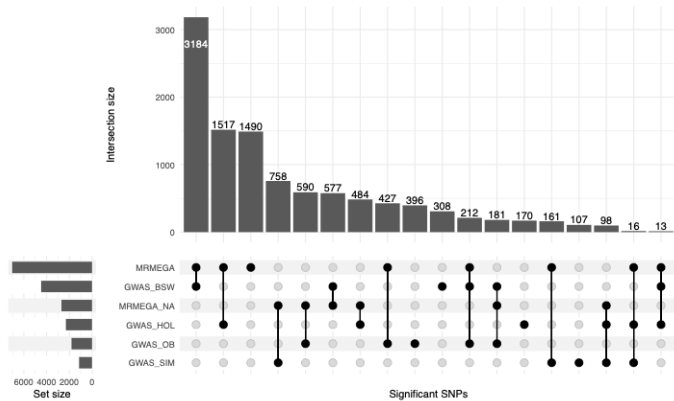

B

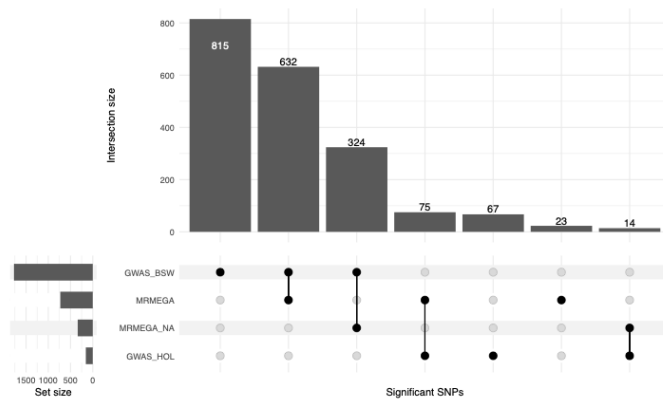

C

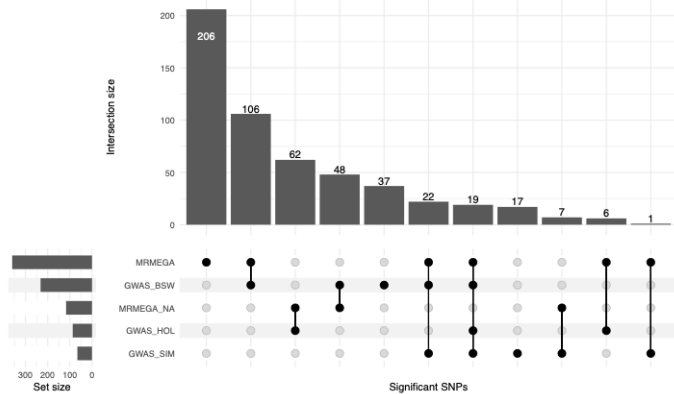

D

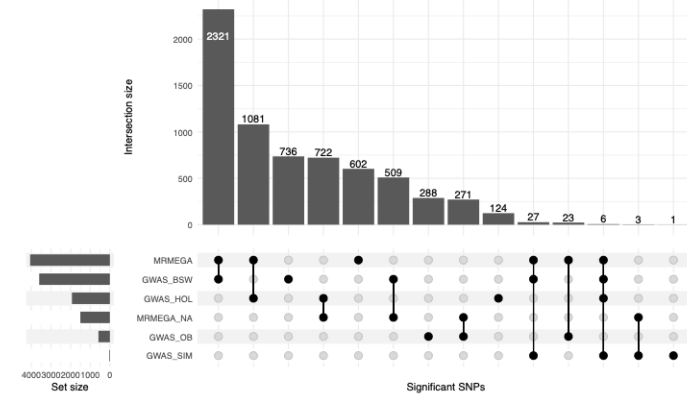

Supplement: Supplementary file 15 — Supplementary Material 15: Figure S14. Overlap of significant SNPs identified by MR-MEGA meta-analysis and single-breed GWAS. Number of shared and unique SNPs surpassing the genome-wide significance threshold (p < 5e−09). Breeds included were Brown Swiss (BSW), Original Braunvieh (OB), Holstein (HOL) and Simmental (SIM). Traits included were A stature (height at sacral bone—KBHM, B fore udder position, C udder central ligament and D front teat position. [file 12711_2026_1042_MOESM15_ESM.pdf]

A

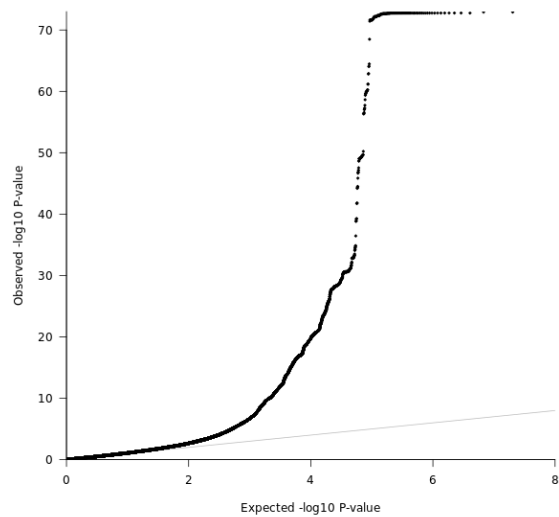

B

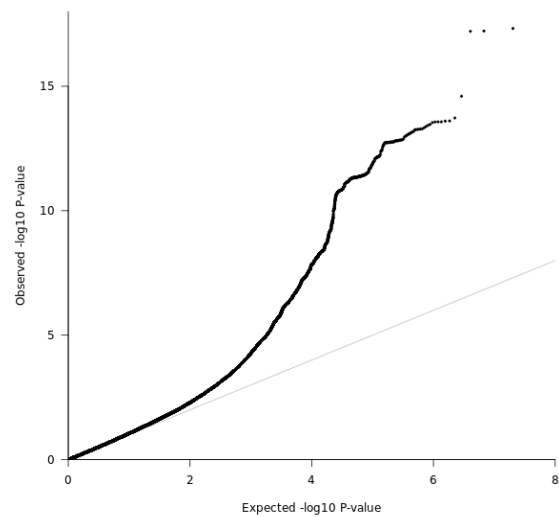

C

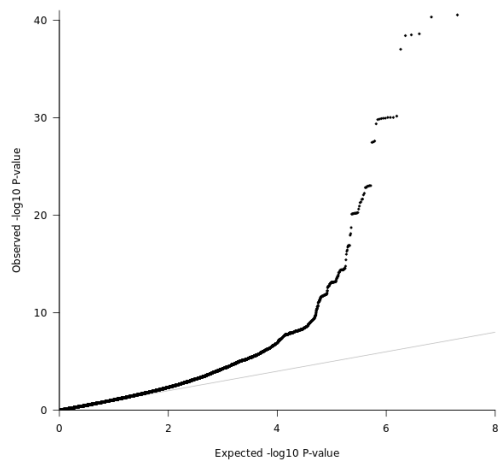

D

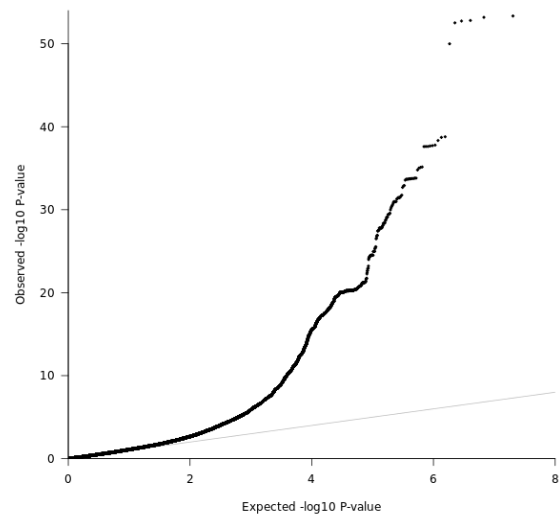

Supplement: Supplementary file 16 — Supplementary Material 16: Figure S15. QQ plots from MR-MEGA meta-analysis. Traits included were A stature (height at sacral bone), B fore udder position, C udder central ligament and D front teat position. The observed –log10(p) values are plotted against the expected distribution under the null hypothesis. [file 12711_2026_1042_MOESM16_ESM.pdf]

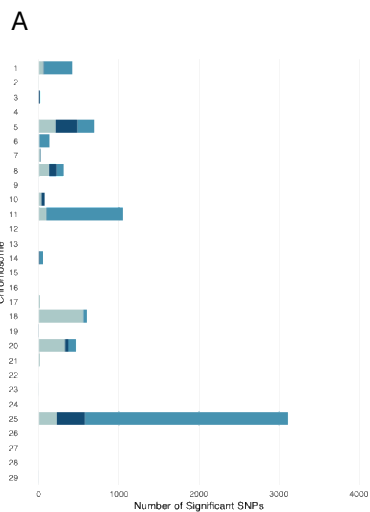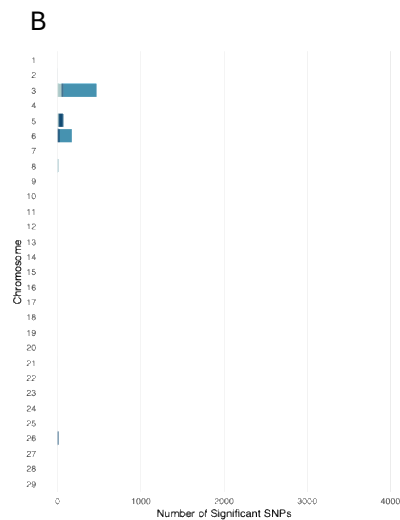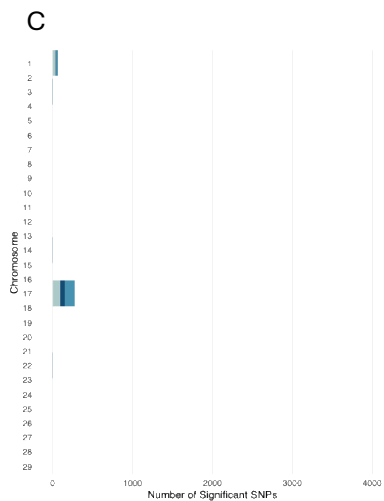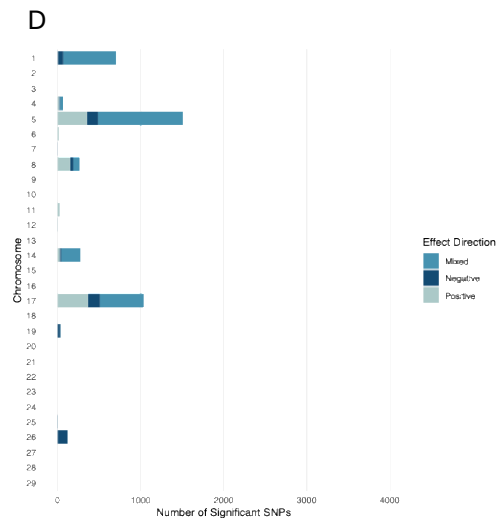

Supplement: Supplementary file 17 — Supplementary Material 17: Figure S16. Effect direction of the significant variants from the MR-MEGA meta-analysis. Number of significant SNPs per chromosome with effect direction identified by MR-MEGA. The observed p value significance threshold is (p < 5e−09). Traits included were A stature (height at sacral bone), B fore udder position, C udder central ligament and D front teat position. [file 12711_2026_1042_MOESM17_ESM.pdf]

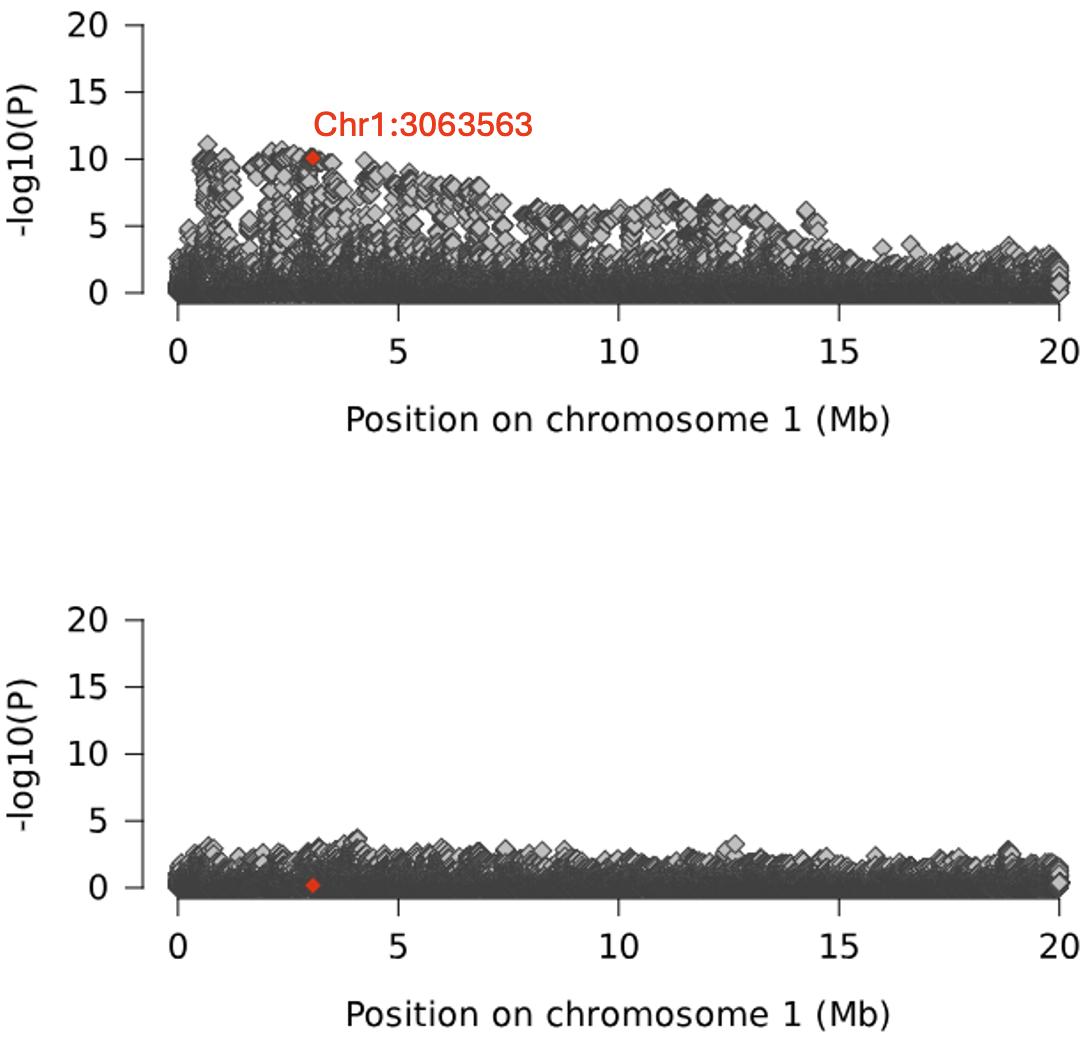

Supplement: Supplementary file 18 — Supplementary Material 18: Figure S17. Association testing for front teat position in Holstein (HOL) cattle with known POLLED genotype. The upper panel shows the results of an association study between imputed sequence variant genotypes on BTA1 and front teat position in 3397 HOL cows with known POLLED genotype. The lower panel shows the results of an association study between imputed sequence variant genotypes on BTA1 and front teat position in 3073 HOL cows not carrying the Friesian polled variant. The red symbol represents the most significantly associated marker from the across-breed meta-analysis. [file 12711_2026_1042_MOESM18_ESM.png]
