# Supplementary material for: Across-breed analyses of genome-wide association studies for stature and mammary gland morphology in cattle reveal pleiotropic effects of the Friesian POLLED haplotype
Source: Genet Sel Evol. 2026 Mar 11;58:19. doi: 10.1186/s12711-026-01042-z (PMC12983537; doi:10.1186/s12711-026-01042-z)
Supplement: Supplementary file 12 — Supplementary Material 12: Figure S11. Significant chromosomes dominance vs additive SNPs for stature. Additive and dominance GWAS results for stature were compared by plotting –log10(p) values from both models for chromosomes showing genome-wide significance under the dominance model. Breeds included were Brown Swiss (BSW), Original Braunvieh (OB), Holstein (HOL) and Simmental (SIM). Variants were classified as significant (p < 5e−09) under the additive model, the dominance model, or both models, and coloured accordingly (additive = dark blue, dominance = teal, both = orange). [file 12711_2026_1042_MOESM12_ESM.pdf]

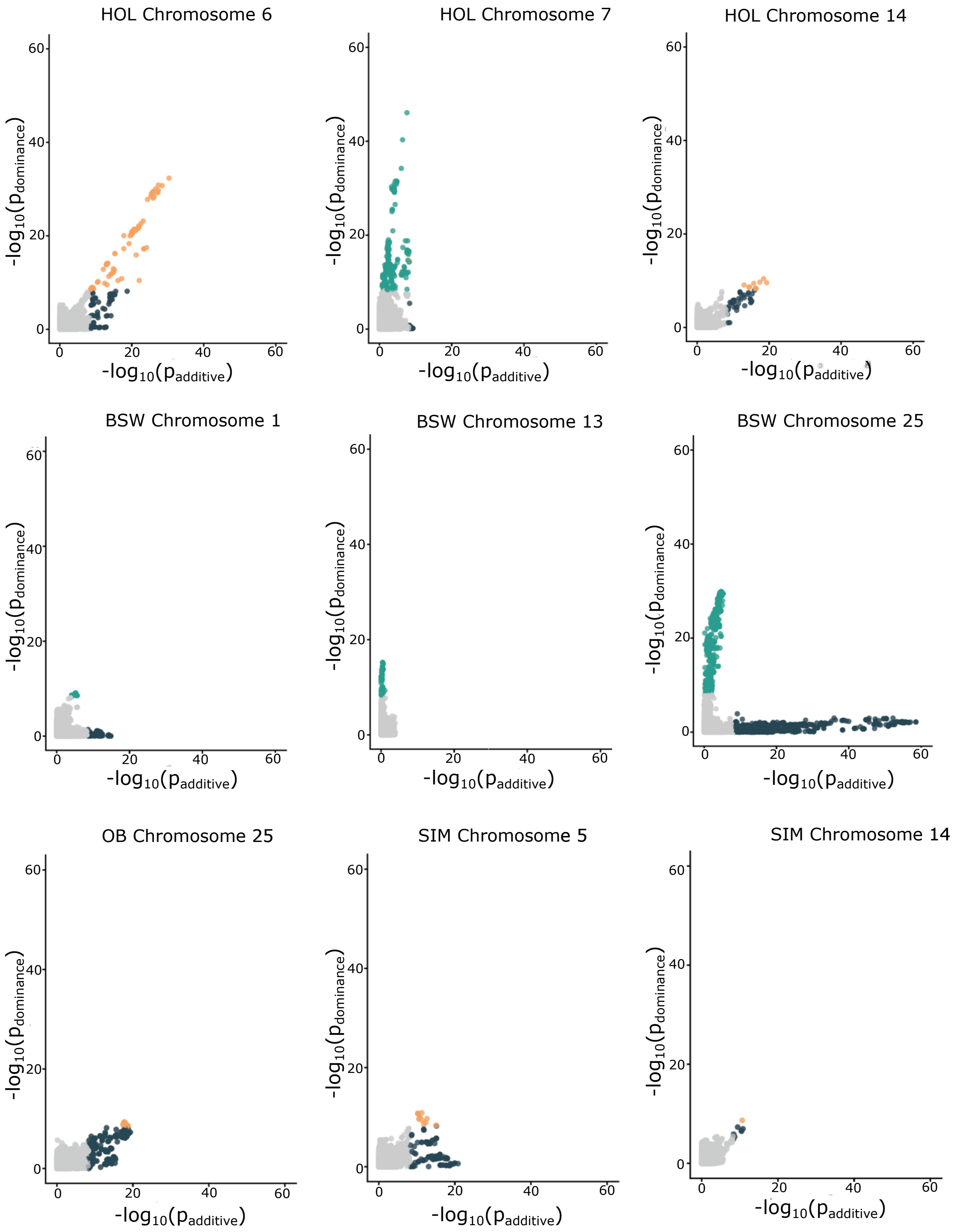

HOL Chromosome 6
HOL Chromosome 7
HOL Chromosome 14
BSW Chromosome 1
BSW Chromosome 13
BSW Chromosome 25
OB Chromosome 25
SIM Chromosome 5
SIM Chromosome 14
-log10(p dominance)
-log10(p additive)
0
20
40
60
